# STRAP Acts as a Scaffolding Protein in Controlling the TLR2/4 Signaling Pathway

**DOI:** 10.1038/srep38849

**Published:** 2016-12-09

**Authors:** Hyunbin D. Huh, Eun A. Ra, Taeyun A. Lee, Sujin Kang, Areum Park, Eunhye Lee, Junhee L. Choi, Eunji Jang, Ji Eun Lee, Sungwook Lee, Boyoun Park

**Affiliations:** 1 Department of Systems Biology, College of Life Science and Biotechnology, Yonsei University, Seoul, South Korea; 2 Department of Health Sciences and Technology, Samsung Advanced Institute for Health Sciences and Technology, Sungkyunkwan University, Seoul, 06351, South Korea; 3 Samsung Genome Institute (SGI), Samsung Medical Center, Seoul, 06351, South Korea; 4 National Research Institute, National Cancer Center, 323 Ilsan-ro, Ilsandong-gu, Goyang-si, Gyeonggi-do 10408, South Korea

## Abstract

The WD40-repeat protein serine/threonine kinase receptor-associated protein (STRAP) is involved in the regulation of several biological processes, including cell proliferation and apoptosis, in response to various stresses. Here, we show that STRAP is a new scaffold protein that functions in Toll-like receptor (TLR)-mediated immune responses. STRAP specifically binds transforming growth factor β-activated kinase 1 (TAK1) and IκB kinase alpha (IKKα) along with nuclear factor-κB (NF-κB) subunit p65, leading to enhanced association between TAK1, IKKα, and p65, and subsequent facilitation of p65 phosphorylation and nuclear translocation. Consequently, the depletion of STRAP severely impairs interleukin-6 (IL-6), tumor necrosis factor alpha (TNF-α), and IL-1β production, whereas its overexpression causes a significant increase in the secretion of these pro-inflammatory cytokines by TLR2 or TLR4 agonist-stimulated macrophages. Notably, STRAP translocates to the nucleus and subsequently binds to NF-κB at later times after lipopolysaccharide (LPS) stimulation, resulting in prolonged IL-6 mRNA production. Moreover, the C-terminal region of STRAP is essential for its functional activity in facilitating IL-6 production. Collectively, these observations suggest that STRAP acts as a scaffold protein that positively contributes to innate host defenses against pathogen infections.

Toll-like receptors (TLRs) are involved in the detection of conserved pathogen components such as pattern-recognition receptors and are important for innate immune responses^1^,^2^. The intracytoplasmic Toll/IL-1 receptor (TIR) domain of TLRs is required for downstream signal transduction because it interacts with myeloid differentiation primary response gene 88 (MyD88) or TIR-domain-containing adapter-inducing interferon-β (TRIF). The MyD88-dependent pathway is common to all TLRs except TLR3, which recruits TRIF as an adaptor protein. In particular, TLR4 is the only known TLR that uses the adaptor proteins of both pathways, thus activation of TLR4 signaling leads to two distinct signaling pathways. TLR2 selectively recognizes a variety of microbial components, such as lipoproteins, lipopeptides, or peptidoglycans, leading to the activation of pro-inflammatory cytokine production. The pro-inflammatory pathway through TLR2 or TLR4 signaling is dependent on the E3 ubiquitin ligase tumor necrosis factor receptor-associated factor 6 (TRAF6), transforming growth factor β-activated kinase 1 (TAK1), and IκB kinase (IKK), which are critical adaptors for the activation and nuclear translocation of nuclear factor-κB (NF-κB), thereby leading to the transcriptional induction of inflammatory genes.

Serine-threonine kinase receptor-associated protein (STRAP) contains seven WD40 domains, which contribute to its regulatory functions in several cellular processes including signal transduction, cell cycle progression, transcription regulation, RNA processing, and vesicular trafficking^3^,^4^,^5^. Particularly, STRAP serves as an inhibitor of transforming growth factor (TGF) signaling by interacting with SMAD7 and the TGF-β receptor^6^. Likewise, STRAP inhibits apoptosis signal-regulating kinase (ASK1)-mediated signaling by stabilizing the formation of a complex between ASK1 and thioredoxin or 14-3-3^7^. In addition, STRAP can activate the phosphoinositide 3-kinase (PI3K) signaling pathway by reducing the association of phosphoinositide-dependent protein kinase-1 (PDK1) with 14-3-3^8^. Stable expression of STRAP also activates the mitogen-activated protein kinase (MAPK)/extracellular signal-regulated kinase (ERK) pathway and downregulates the cyclin-dependent kinase (CDK) inhibitor p21 Cip1^3^,^9^. Moreover, STRAP binds to upstream of N-ras (Unr) and gem-associated protein 7 (Gemin7), which are involved in the regulation of cap-independent translation and the assembly of small nuclear ribonucleoproteins (snRNPs)^10^,^11^. Although STRAP has multiple biological functions, the involvement of STRAP in the TLR signaling pathway has not yet been determined.

Here, we propose that STRAP acts as a scaffold protein for NF-κB p65 activation through its interaction with TAK1, IKKα, and p65 in the early phase following lipopolysaccharide (LPS) stimulation, and that the nuclear localization of STRAP is involved in the prolonged activation of NF-κB at later time points after LPS stimulation. Moreover, the C-terminal region of STRAP, is necessary to enhance p65 phosphorylation, its interaction with TAK1, and subsequently the production of pro-inflammatory cytokines. Overall, these findings suggest the existence of a new scaffold protein that positively and continually regulates pro-inflammatory cytokine gene expression during pathogen infection.

## Results

### STRAP enhances pro-inflammatory cytokine production in the TLR-mediated innate immune response

**Figure 1:**
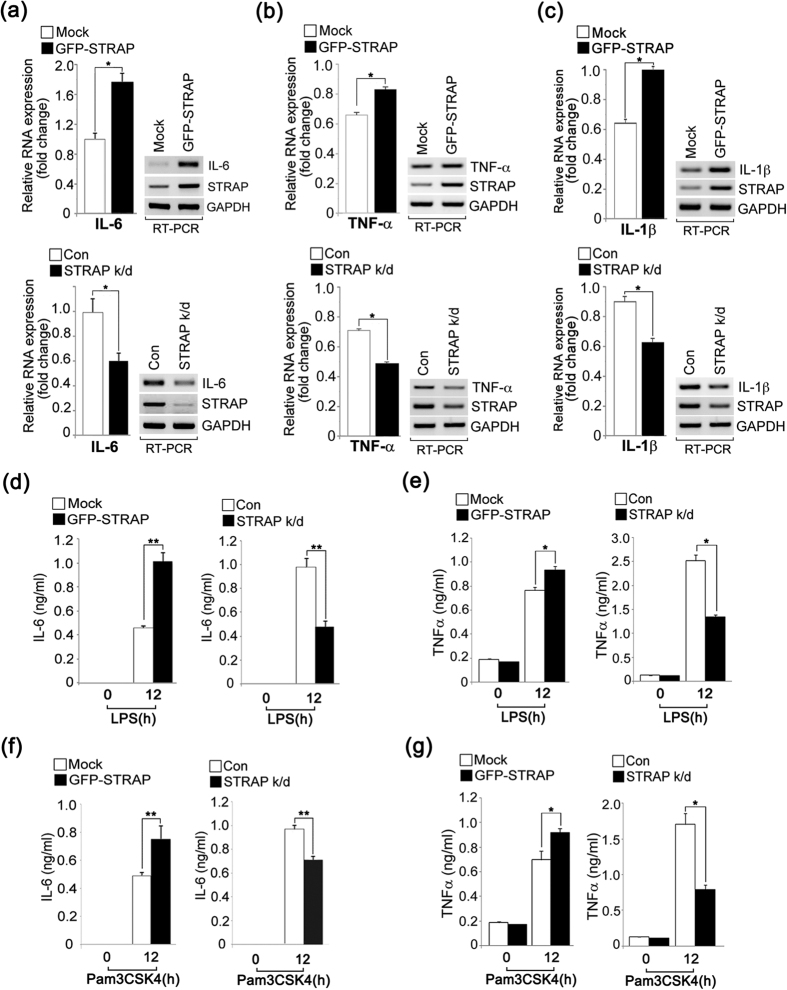
STRAP upregulates TLR-mediated pro-inflammatory cytokine production. (**a–c**) Overexpression of STRAP enhances IL-6, TNF-α, and IL-1β mRNA, whereas STRAP deficiency diminished those mRNA levels in LPS-treated RAW 264.7 macrophages. Cells stably expressing shRNA-GFP (control), shRNA-STRAP (STRAP k/d), vector (Mock), or GFP-STRAP (GFP-STRAP) were treated with LPS (80 ng/ml) for 12 h. The IL-6, TNF-α, or IL-1β mRNA levels were analyzed by RT-PCR. Expression was quantified by densitometry of bands and reported relative to GAPDH. *P < 0.01 (Student’s *t*-test). (**d–g**) IL-6 and TNF-α production is affected by STRAP expression levels in LPS- or Pam3CSK4-stimulated RAW macrophages. Cells were treated with LPS (80 ng/ml) or Pam3CSK4 (100 ng/ml) for 12 h, respectively. IL-6 and TNF-α levels were analyzed by ELISA. *P < 0.01 and **P < 0.005 (Student’s *t*-test). Data are representative of three independent experiments and are presented as mean ± s.d. in (**a–g**).

Several WD-motif containing proteins, such as WDR36, WDR62, and Morg1 are involved in protein-protein interactions and participate in different biological functions^12^,^13^,^14^,^15^. In particular, STRAP plays a role in regulating the TGF-β signaling pathway, which is involved in cross-talk with the NF-κB signaling pathway^6^,^16^. We therefore hypothesized that STRAP might play a role in the TLR-mediated immune response. To study the possible role(s) of STRAP in the TLR signaling pathway, we first investigated the ability of STRAP to induce IL-6 production in response to LPS, which promotes robust cytokine production and induces innate immunity. To test this, we generated RAW 264.7 macrophages stably expressing green fluorescence protein (GFP)-tagged STRAP or a short hairpin RNA (shRNA) targeting STRAP. Following LPS stimulation, IL-6 mRNA levels were increased in GFP-STRAP-overexpressing cells (Fig. 1a**, upper graph** and **panels**). Conversely, the depletion of STRAP impaired the IL-6 mRNA levels after stimulation with LPS (Fig. 1b**, bottom graph** and **panels**). We also examined two other pro-inflammatory cytokines, TNF-α or IL-1β, in STRAP-overexpressing or -depleted macrophages with LPS stimulation. The production of these cytokines was similarly significantly affected by STRAP expression levels (Fig. 1b and c).

We investigated whether the effects on IL-6 and TNF-α protein levels were similar in STRAP-overexpressing and STRAP-depleted macrophages, and found that IL-6 expression was enhanced by GFP-STRAP overexpression but was reduced by shRNA-STRAP compared to control cells (Fig. 1d and e). To assess the effect of TLR-agonist specificity on STRAP-dependent IL-6 or TNF-α production, we measured its production in macrophages exposed to the synthetic triacylated lipoprotein Pam3CSK4, which activates the TLR2 signaling cascade. Similar to results seen with LPS stimulation, under Pam3CSK4-stimulated conditions, IL-6 or TNF-α levels were also significantly affected by STRAP-overexpression or -depletion (Fig. 1f and g). Therefore, these results suggest that STRAP enhances both the TLR2- and TLR4-mediated signaling pathways.

### STRAP facilitates NF-κB phosphorylation and nuclear translocation

**Figure 2:**
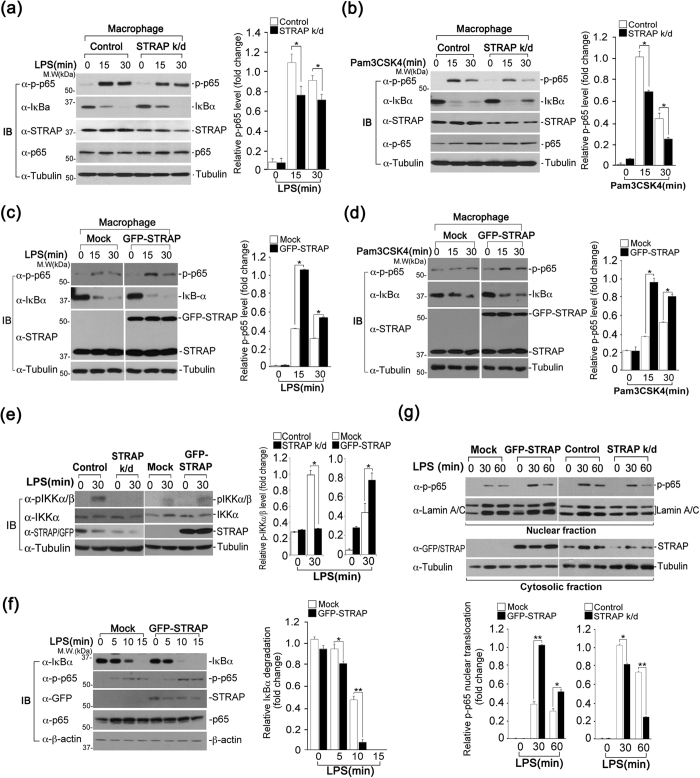
STRAP enhances phosphorylation of NF-κB p65. (**a–d**) STRAP impacts the activation of p65. Phosphorylation of p65 was analyzed by immunoblotting in LPS-stimulated RAW macrophages stably expressing shRNA-GFP (control) or shRNA-STRAP (STRAP k/d) (**a,b**), and in mock-transfected or GFP-STRAP-expressing LPS-stimulated RAW macrophages (**c,d**). Expression levels of phosphorylated p65 were quantified by densitometry of bands and reported relative to tubulin. (**e**) The expression level of STRAP affects IKKα phosphorylation in response to LPS. Phospho-IKKα levels in STRAP-overexpressed or -depleted cells were determined by immunoblotting with anti-phospho-IKKα antibody. Expression levels of phosphorylated IKKα were quantified by densitometry of bands and reported relative to tubulin. (**f**) STRAP overexpression enhances a more rapid degradation of IκBα. The kinetics of IκBα degradation in STRAP-overexpressed or -depleted cells was determined by immunoblotting with anti-IκBα antibody. Expression levels of IκBα were quantified by densitometry of bands and reported relative to β-actin. (**g**) The nuclear translocation of phospho-p65 (p-p65) was influenced by STRAP expression levels. Expression of both STRAP and p-p65 was analyzed in the cytosolic and nuclear fractions of LPS-stimulated cells stably expressing GFP-STRAP or shRNA-STRAP (STRAP k/d) by immunoblotting. Tubulin and lamin A/C were used as loading controls for the cytosolic and nuclear fractions, respectively. Nuclear translocation levels of phosphorylated p65 were quantified by densitometry of bands and reported relative Lamin A/C. *P < 0.01 and **P < 0.005 (Student’s *t*-test). Data are representative of three independent experiments and are presented as mean ± s.d. in (**a–g**).

To identify the molecular functions of STRAP in the TLR2 and TLR4-mediated signaling pathways, we investigated the effect of STRAP on the phosphorylation of NF-κB subunit p65. Notably, the phosphorylation of NF-κB p65 was reduced at 15 min or 30 min after LPS stimulation in STRAP-depleted macrophages, compared to cells stably expressing shRNA-GFP (control) (Fig. 2a). We also assessed the effect of STRAP depletion on the phosphorylation of p65 in response to Pam3CSK4. Similarly, phospho-p65 levels were decreased in STRAP-depleted cells at 15 min or 30 min after Pam3CSK4 stimulation (Fig. 2b). To confirm STRAP-dependent phosphorylation of p65, we examined the effect of GFP-STRAP overexpression on NF-κB p65 phosphorylation. GFP-STRAP-overexpressing cells exhibited a considerable increase in NF-κB p65 phosphorylation 15 min or 30 min after LPS or Pam3CSK4 stimulation, compared with control cells transfected with the empty (Mock) vector (Fig. 2c and d).

Next, we sought to determine whether the reduced p65 phosphorylation is due to decreased activation of IKKα, a critical kinase for phosphorylating p65. Because the phosphorylated form of IKKα (induced by TAK1) is active in inducing p65 phosphorylation, we examined IKKα phosphorylation in STRAP-depleted or -overexpressing cells. We observed that IKKα phosphorylation was considerably decreased upon STRAP depletion (Fig. 2e). In contrast, a significant increase in IKKα phosphorylation was observed in STRAP-overexpressing macrophages (Fig. 2e). In addition, STRAP overexpression induced a more rapid degradation of IκBα in response to LPS (Fig. 2f). Moreover, the translocation of phosphorylated endogenous p65 to the nucleus was considerably enhanced in GFP-STRAP-expressing macrophages 30 min or 60 min after LPS stimulation (Fig. 2g). On the contrary, the nuclear translocation of p65 was reduced in STRAP-depleted cells (Fig. 2g). Taken together, these results suggest that STRAP enhances NF-κB p65 phosphorylation as well as its nuclear translocation.

### STRAP acts as a scaffold protein in the TAK1-IKKα-p65 signaling cascade

**Figure 3:**
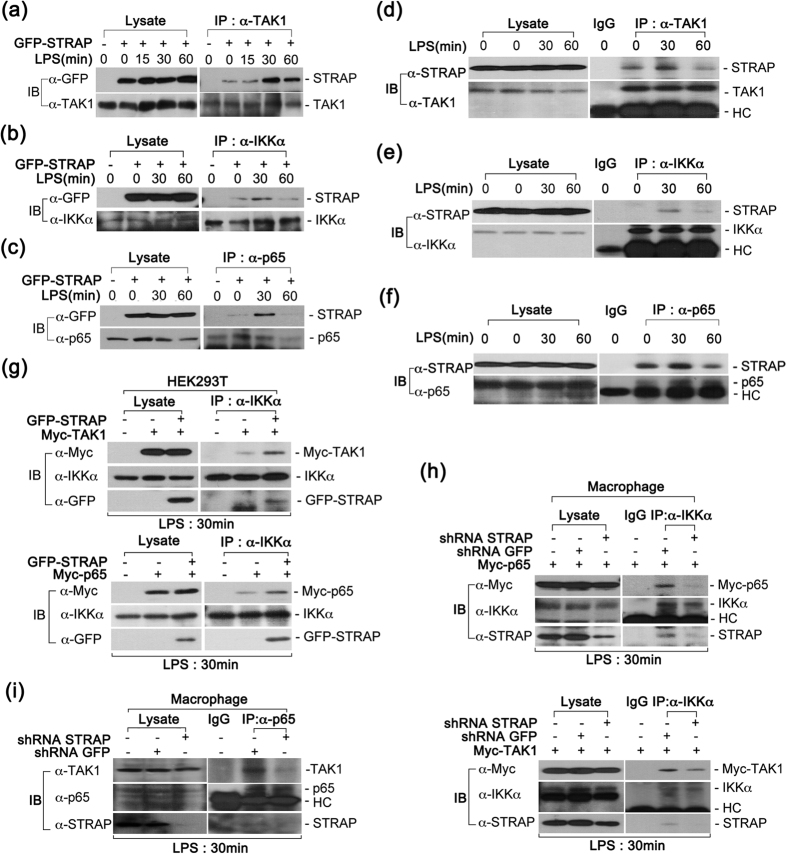
STRAP associates with TAK1, IKKα, and p65. (**a–c**) STRAP interacts with TAK1, IKKα, and p65. RAW 264.7 cells stably expressing GFP-STRAP were treated with LPS (80 ng/ml) at different time points. Cell lysates were immunoprecipitated with anti-TAK1 (**a**), anti-IKKα (**b**), or anti-p65 (**c**) antibodies and immunoblotted with anti-GFP antibody. (**d–f**) STRAP endogenously binds to TAK1, IKKα, and p65. After LPS stimulation, cell lysates were immunoprecipitated with anti-TAK1 (**d**), anti-IKKα (**e**), or anti-p65 (**f**) antibodies and immunoblotted with anti-STRAP antibody. (**g**) The interaction between IKKα and TAK1 or IKKα and p65 was increased by STRAP overexpression. The association of IKKα with TAK1 or p65 was determined by immunoprecipitation with anti-IKKα antibody and immunoblotting with the indicated antibodies. (**h**) The interaction of IKKα with p65 or TAK1 was reduced by STRAP depletion. Lysates from LPS-stimulated RAW macrophages stably expressing shRNA-GFP or shRNA-STRAP were immunoprecipitated with anti-IKKα antibody and immunoblotted with the indicated antibodies. (**i**) TAK1 endogenously interacts with p65 and its interaction is reduced by STRAP depletion. Lysates from LPS-stimulated macrophages were immunoprecipitated with anti-p65 antibody and immunoblotted with anti-TAK1 antibody. Data are representative of three independent experiments.

The binding of TLR2/4 with their ligands subsequently triggers the activation of the downstream kinases TAK1 and IKKα, which is essential for facilitating NF-κB p65 phosphorylation^17^. Because STRAP contains seven WD40-repeat domains, we hypothesized that STRAP might act as a scaffold protein and facilitate the recruitment of critical kinases, leading to the activation of p65 phosphorylation. To explore this possibility, we examined the interaction of STRAP with TAK1 and IKKα in LPS-stimulated macrophages. Following LPS stimulation, the lysates from macrophages stably expressing GFP-STRAP were immunoprecipitated using anti-TAK1 antibody and subjected to immunoblotting with an anti-GFP antibody. Interestingly, the relatively high-affinity interaction of STRAP with TAK1 was clearly detected at 30 min (Fig. 3a). Consistent with the TAK1 results, STRAP also bound IKKα, and the interaction likewise peaked at 30 min and decreased 60 min after LPS stimulation (Fig. 3b). Moreover, the association of STRAP with NF-κB p65 was similar to the binding of STRAP to TAK1 and IKKα (Fig. 3c). To confirm these results, we assessed these interactions at endogenous levels. Consistently, we clearly observed that endogenous STRAP interacted with endogenous TAK1, IKKα, and p65 (Fig. 3d,e and f).

We next investigated whether the TAK1-IKKα interaction was affected by STRAP expression. Notably, the interaction of TAK1 with IKKα was increased in GFP-STRAP- expressing cells when compared to control cells (Fig. 3g**, upper panels**). Similar to the TAK1-IKKα binding results, STRAP-overexpressing cells clearly exhibited an increase in the binding of IKKα with NF-κB p65 (Fig. 3g**, bottom panels**). Moreover, the interaction of IKKα with p65 or TAK1 was significantly reduced upon STRAP depletion (Fig. 3h). We also confirmed that the binding of endogenous TAK1 to p65 was clearly decreased in STRAP-depleted macrophages (Fig. 3i). Taken together, these results suggest that STRAP functions as a scaffold protein capable of binding TAK1, IKKα, and p65, thereby enhancing the TAK1-IKKα or IKKα-p65 interactions and subsequently promoting phosphorylation and nuclear translocation of NF-κB p65.

### Nuclear translocation of STRAP is associated with prolonged NF-κB activity

**Figure 4:**
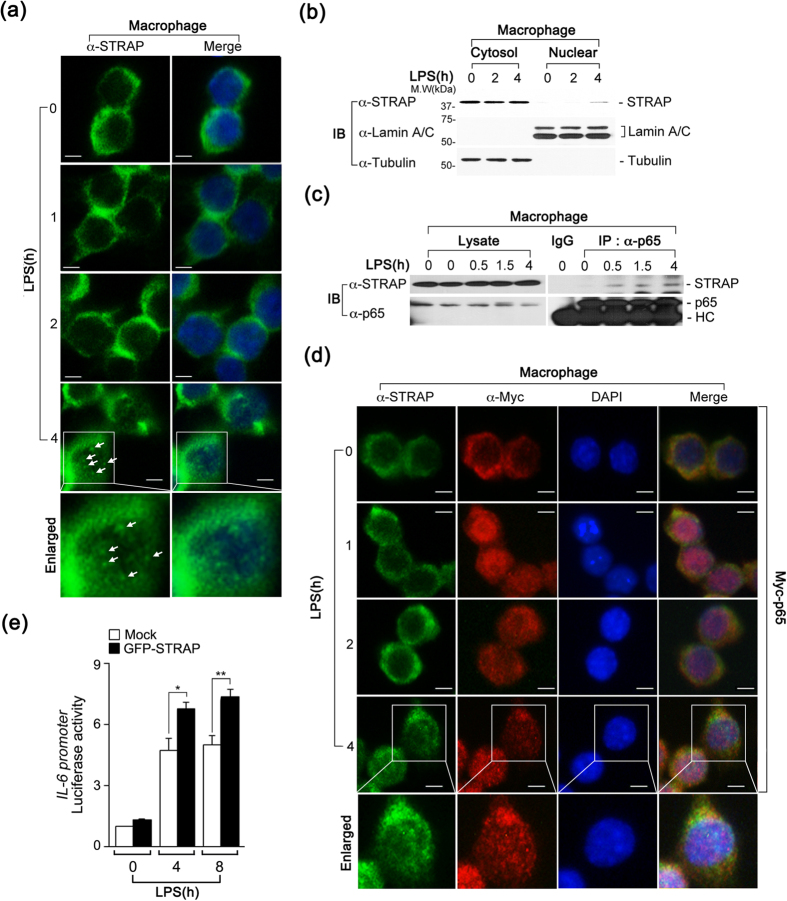
Nuclear localization of STRAP is linked to NF-κB activation during the late phase of LPS-stimulation. (**a**) LPS induces the translocation of STRAP to the nucleus at 4 h. STRAP was examined by an immunofluorescence assay (IFA) using an anti-STRAP antibody, and the nuclei were stained with 4′,6-diamidino-2-phenylindole (DAPI). Scale bars, 5 μm. (**b**) STRAP translocates to the nucleus in response to LPS at 4 h. Endogenous STRAP was analyzed in the cytosolic and nuclear fractions of LPS-stimulated RAW cells by immunoblotting. Tubulin and lamin A/C were used as loading controls for the cytosolic and nuclear fractions, respectively. (**c**) STRAP endogenously interacts with p65 in response to LPS at 4 h. RAW cells were treated with LPS (80 ng/ml) at different time points. Cell lysates were immunoprecipitated with anti-p65 antibody and immunoblotted with anti-STRAP or anti-p65 antibodies. (**d**) STRAP and p65 localizes to the nucleus at 4 h following LPS-stimulation. STRAP and Myc-p65 were visualized by IFA with anti-STRAP and anti-Myc antibodies, and the nuclei were stained with DAPI. Scale bars, 5 μm. (**e**) STRAP enhances the transcriptional activity of IL-6. Mouse embryonic fibroblasts (MEFs) were transfected with an empty vector (Mock) or GFP-STRAP vector (GFP-STRAP) along with the IL-6 luciferase reporter and Renilla reporter and incubated with LPS (80 ng/ml) for 4 h. *P < 0.01 and **P < 0.005 (Student’s *t*-test). Data are representative of three independent experiments and are presented as mean ± s.d. in (**e**).

STRAP is known to localize primarily in the cytosol, where it interacts with binding partners and regulates a variety of biological functions. Hence, we next investigated whether LPS stimulation affected the subcellular localization of STRAP. In accord with previous findings, we observed the cytosolic localization of STRAP at early time points (0, 1, and 2 h) following LPS stimulation (Fig. 4a). Interestingly, STRAP was partially enriched within the subnuclear foci at 4 h after LPS stimulation (Fig. 4a**, arrows**). To confirm these results, a biochemical fractionation was performed on RAW macrophages in response to LPS. In cells incubated with LPS for 4 h, endogenous STRAP was clearly observed in the nuclear fraction and the expression patterns were similar to those observed in the immunofluorescence assay (IFA) data (Fig. 4b**, first panel**). The presence and purity of the cytosolic and nuclear fractions were determined using the cytosolic and nuclear markers, α-tubulin and lamin A/C, respectively (Fig. 4b**, second** and **third panels**).

To understand the effect of the nuclear localization of STRAP on NF-κB in response to LPS, we examined the interaction of STRAP with NF-κB p65 at later time points following LPS stimulation. We previously noted that the interaction of STRAP with p65 peaked at 30 min and subsequently decreased at 60 min, which was similar to the binding pattern apparent between STRAP and TAK1 or IKKα (Figs 3a~f). Interestingly, the observed binding of STRAP to p65 was strong at 4 h after LPS stimulation, following the translocation of STRAP to the nucleus (Fig. 4c). Furthermore, a merged image of STRAP and Myc-p65 revealed that these proteins partially overlapped in the nucleus at 4 h in LPS-stimulated macrophages (Fig. 4d). To determine whether nuclear import of STRAP is linked to an increase in NF-κB promoter activity, we transfected cells with an *il-6*–luciferase reporter gene with or without GFP-STRAP at different time points following LPS stimulation and noted that GFP-STRAP-expressing cells exhibited enhanced luciferase activity compared to control cells (Fig. 4e). Taken together, these data demonstrate that nuclear translocation of STRAP is involved in prolonged activation of NF-κB p65 in the late phase following LPS stimulation.

### The C-terminal domain of STRAP is essential for enhanced IL-6 production

**Figure 5:**
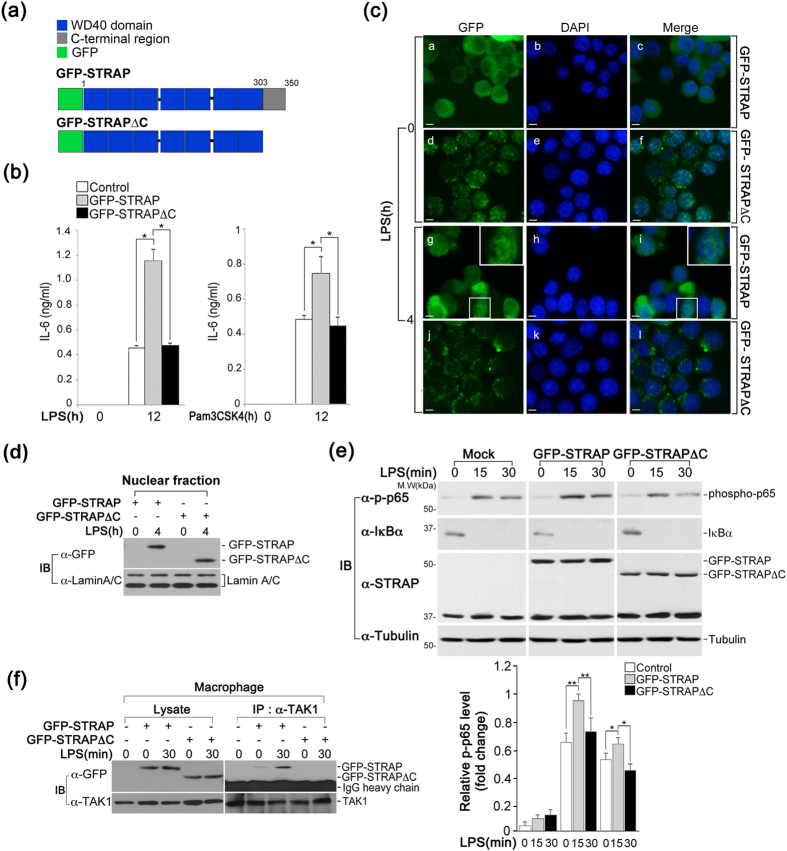
The C-terminus of STRAP is critical for IL-6 expression. (**a**) Schematic model of STRAP and its C-terminal deletion mutant (GFP-STRAP ΔC) tagged with GFP. (**b**) GFP-STRAP ΔC is not sufficient to significantly enhance IL-6 production, in contrast to wild-type STRAP. RAW macrophages stably expressing Mock, GFP-STRAP, or GFP-STRAP ΔC were treated with LPS (80 ng/ml) or Pam3CSK4 (100 ng/ml) for 12 h. IL-6 levels were analyzed by ELISA. *P < 0.01. (**c**) The C-terminus of STRAP is involved in its subcellular localization. Macrophages stably expressing GFP-STRAP or GFP-STRAP ΔC were examined by IFA, and nuclei were stained with DAPI. Scale bars, 5 μm. (**d**) Nuclear translocation of GFP-STRAP ΔC in response to LPS. The presence of GFP-STRAP and GFP-STRAP ΔC in the nuclear fractions of LPS-stimulated RAW cells was analyzed by immunoblotting. Lamin A/C was used as loading controls for the nuclear fractions. (**e**) GFP-STRAPΔC was not as effective in promoting p65 phosphorylation as wild-type STRAP. The phosphorylation of p65 was analyzed in LPS-stimulated RAW cells stably expressing Mock, GFP-STRAP, or GFP-STRAP ΔC by immunoblotting. Expression levels of phosphorylated p65 were quantified by densitometry of bands and reported relative to tubulin. *P < 0.01 and **P < 0.005. (**f**) The C-terminus of STRAP is important for its association with TAK1. RAW cells stably expressing GFP-STRAP or GFP-STRAP ΔC were treated with LPS (80 ng/ml). Cell lysates were immunoprecipitated with an anti-TAK1 antibody and immunoblotted with anti-GFP or anti-TAK1 antibodies. Data are representative of three independent experiments and are presented as mean ± s.d. in (**b**,**e**).

Previous reports have shown that the C-terminal region of STRAP contains serine/threonine/tyrosine phosphorylation sites and is required for its binding with other phosphoproteins^6^,^9^,^18^. To determine whether the C-terminus of STRAP contributes to the TLR-triggered signaling pathway, we analyzed IL-6 production in LPS-stimulated macrophages expressing a C-terminal GFP-STRAP deletion mutant (GFP-STRAPΔC) that entailed a deletion from the carboxyl terminus to amino acid 303 (Fig. 5a). In accordance with previous results, wild-type STRAP-overexpressing cells following stimulation with LPS showed significantly greater IL-6 production than seen in control cells; however, no differences in IL-6 production were observed in GFP-STRAPΔC-expressing macrophages compared to control cells, suggesting that GFP-STRAPΔC was not as effective in promoting IL-6 production as wild-type STRAP (Fig. 5b**, left graph**). The same effect was observed in cells expressing GFP-STRAPΔC following stimulation with Pam3CSK4 (Fig. 5b**, right graph**). Furthermore, the subcellular location pattern of GFP-STRAPΔC revealed an unusually coarse and diffuse cytosolic distribution with sparse punctate spots, which was not affected by LPS stimulation (Fig. 5c); however, larger amounts of GFP-STRAPΔC were observed in the nuclear fractions in a pattern similar to that of wild-type STRAP (Fig. 5d). These results suggest that the C-terminal region of STRAP is necessary to maintain its precise subcellular distribution and enhance the production of IL-6.

We next investigated whether the C-terminal region of STRAP affects p65 phosphorylation and its interaction with TAK1. The increase in p65 phosphorylation in GFP-STRAPΔC-expressing macrophages was not as high as that seen in the case of wild-type STRAP, but was instead similar to that seen in control cells (Fig. 5e). Furthermore, the ability of STRAP to bind TAK1 was clearly impaired by the deletion of its C-terminal region (Fig. 5f). Taken together, these findings suggest that the C-terminal region of STRAP is required for its functional activity in the TLR signaling pathway.

## Discussion

**Figure 6:**
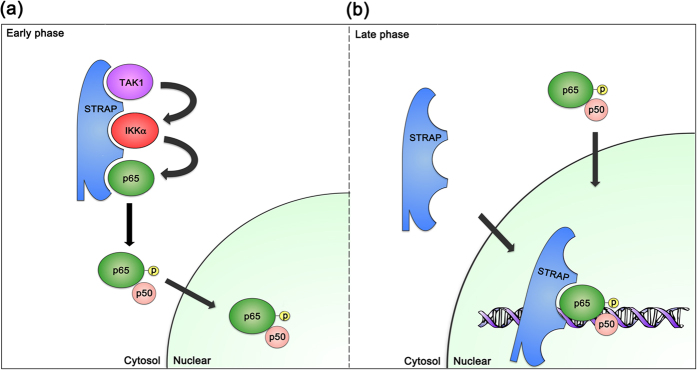
Model of STRAP-mediated NF-κB activation in the TLR signaling pathway. Following TLR stimulation, STRAP interacts with signaling regulators (TAK1, IKKα, and p65) as a scaffold protein in the early phase, and enters the nucleus in the late phase of TLR signaling to activate NF-κB-dependent genes.

We report that STRAP serves as a scaffold protein and positively regulates the TLR-mediated signaling pathway. To this end, STRAP provides a platform for the binding of TAK1, IKKα, and NF-κB subunit p65, which is essential for facilitating p65 phosphorylation in response to LPS stimulation. Moreover, STRAP is transported to the nucleus at later time points following LPS stimulation and thereby facilitates the prolonged activation of NF-κB. In addition, the C-terminus of STRAP is necessary for its interaction with TAK1, and for NF-κB p65 phosphorylation and the subsequent production of IL-6 in the TLR signaling pathway. Collectively, these results demonstrate that as a scaffold protein, STRAP is essential for the assembly of critical kinases (TAK1 and IKKα) in the early TLR signaling phase and that the nuclear translocalization of STRAP is involved in the prolonged activation of NF-κB p65 in the later phases of TLR signaling (Fig. 6).

As previously noted, STRAP negatively regulates the TGF-β signaling pathway^6^. In the context of the tumor microenvironment, TGF-β exerts tumor suppressor activity through inhibiting cell growth, inducing apoptosis, and down-regulating the *c-Myc* oncogene^19^,^20^,^21^. In addition, NF-κB-mediated inflammation by the activation of TLRs has long been highly linked to tumor progression and metastasis in various cancers^22^. Indeed, the attenuation of TGF-β signaling and aberrant activation of NF-κB are observed in a major subset of early premalignant lesions as well as in head and neck squamous cell carcinoma (HNSCC)^16^,^23^. Furthermore, STRAP is aberrantly overexpressed in colorectal, lung, and breast cancers^9^,^24^. Additionally, the overexpression or knockdown of STRAP increases or decreases tumorigenesis, respectively, in athymic nude mice^9^. Therefore, based on our findings, it is also possible that the aberrant expression or misregulation of STRAP might affect NF-κB activation, along with suppression of TGF-β signaling, thereby potentially contributing to the development of various cancers.

WD40 domains do not possess intrinsic enzymatic activity, but they mediate diverse protein–protein interactions. Therefore, the interactions of these proteins perform a regulatory function in various cellular processes through their scaffolding roles in both the assembly and regulation of dynamic multi-subunit complexes^25^,^26^. For example, the WD40 protein Han11 directly binds HIPK2, MEKK1, and DYRK1a/b as a scaffold protein and regulates kinase signaling^27^, and the WD40 protein Morg1 interacts with both Par6 and Crb3, which not only facilitates Par6 binding to Crb3 but also leads to apical targeting of Par6-aPKC^15^. Thus, the WD40 domains of STRAP might be important for its role as a scaffold protein in the interaction of STRAP with TAK1 and IKKα in TLR-mediated immune responses, and it is possible that STRAP might recruit other cellular proteins in TLR-triggered signaling pathways. In addition, in contrast with STRAP-mediated positive regulation in the MyD88-dependent TLR 2/4 signaling pathway, STRAP may bind to certain adaptor proteins through the WD domain in TRIF-mediated TLR3/4 signaling, distinctly regulating its response in a manner similar to that by which the STRAP-Smad7 interaction causes a synergistic effect in the suppression of TGF-β signaling.

Interestingly, although the STRAP C-terminal deletion mutant (STRAPΔC) contained all seven of the WD40 repeat domains, it was not sufficient to significantly promote NF-κB p65 phosphorylation and IL-6 production to the extent seen with wild-type STRAP. Previously, it was shown that the phosphorylation of STRAP is crucial for its role in cell death through the ASK1, TGF-β, p53, and PI3K/PDK1 signaling pathways^28^. Additionally, rapid induction of STRAP phosphorylation at the tyrosine 342 residue was observed in mast cell signaling^18^. Because STRAP interacts with various kinases, such as ASK1, PDK1, ERK, TAK1, and IKKα, it is possible that the various putative phosphorylation sites of STRAP in the C-terminal region may be involved in its functional activity through specific interactions, leading to regulating the TLR or TGF-β signaling pathways.

Collectively, the findings of the present study reveal a positive regulatory role of STRAP in TLR signaling, which could provide additional insight into the potential prognostic and therapeutic implications of STRAP in various forms of cancer.

## Materials and Methods

### Reagents

LPS (*Escherichia coli*, serotype 026: B6) and Pam3CSK4 were purchased from Sigma-Aldrich (St. Louis, MO) and InvivoGen (San Diego, CA), respectively. DharmaFECT™ 1 (T-2001-01) and OmicsFect (CP2101) were obtained from Dharmacon (Lafayette, CO) and Omics Bio (Taipei City, Taiwan), respectively.

### Antibodies

The following antibodies were used: anti-STRAP(611346, BD Biosciences, San Jose, CA), anti-TAK1(sc-7162, Santa Cruz Biotechnology, Santa Cruz, CA), anti-p-p65(3033,Cell Signaling Technology, Danvers, MA), anti-p65 (sc109, Santa Cruz Biotechnology), anti-IKKα (sc-7218, Santa Cruz Biotechnology), anti-p-IKKα/β (2697, Cell Signaling Technology), anti-IκBα (sc-371, Santa Cruz Biotechnology), anti-Myc (2276, Cell Signaling Technology), anti-GFP (sc-9996, Santa Cruz Biotechnology), anti-Tubulin (G094, ABM Inc, Richmond, Canada), and anti-Lamin A/C (2032, Cell Signaling Technology).

### DNA constructs

Mouse STRAP and STRAP ΔC were tagged with GFP and subcloned into the retroviral pLHCX vector (Clontech, Mountain View, CA). Mouse p65 was fused to Myc and subcloned into the retroviral pMSCV vector (Myc-p65). The shRNA oligonucleotides against STRAP or GFP (control) were annealed and subcloned into the pSUPER retroviral vector (Oligoengine, Seattle, WA) using the following primers: shRNA-GFP (negative control), 5′-GATCCGCAAGCTGACCCTGAAGTTCCTCGAGGAACTTCAGGGTCAGCTTGCTTTTTTGGAAA-3′ (forward) and 5′-AGCTTTTCCAAAAAAGCAAGCTGACCCTGAAGTTCCT CGAGGAA CTTCAGGGTCAGCTTGCG-3′ (reverse); shRNA STRAP, 5′-GATCCGGGTGCAACATTGAATAAGCTCGAGCTTATTCAATGTTGCACCCTTTTTTGGAAA-3′(forward) and 5′-AGCTTTTCCAAAAAAGGGTGCAACATTGA ATAAGCTCGAGCTTATTCAATGTTGCACCCG-3′ (reverse). The following oligonucleotides were used for cloning : GFP-STRAP, 5′-ATTCTCGAGCATGGCCATGAGGCAGACGCC-3′ (forward) and 5′-ATTATCGATTCAGGCCTTAACTTCAGGAGTTGATG-3′ (reverse); GFP- STRAPΔC, 5′-ATTCTCGAGCATGGCCATGAGGCAGACGCC-3′ (forward) and 5′-ATTATCGATTCACAGGCCATAGGTCTTTCCTACCA-3′ (reverse); Myc-TAK1, 5′-ATTCTCGAGATGTCGACAGCCTCCGCCG-3′ (forward) and 5′-ATTGAATTCTCATGAAGTGCCTTGTCGTTTCTGC-3′ (reverse); Myc-p65, 5′-ATCCTCGAGGACGATCTGTTTCCCCTCATCTTTC (forward) and 5′-ATC GAATTCTTAGGAGCTGATCTGACTCAAAAGAGCA (reverse); IL-6 promoter, 5′- AATGAGCTCGCTGACCTAGTATGTACACTAAGTCCA-3′ (forward) and 5′- AATAGATCTAGCGGTTTCTGGAATTGACTATCGTTC-3′ (reverse). All constructs were verified by sequencing.

### Cell culture

Murine RAW 264.7 macrophages (ATCC TIB-71), human embryonic kidney (HEK) 293 T cells (ATCC CRL-11268), and mouse embryonic fibroblasts (MEFs) were grown in Dulbecco’s Modified Eagle Medium (DMEM; HyClone™, Logan, UT) containing 7.5% fetal bovine serum (FBS; Hyclone™) and penicillin/streptomycin (Hyclone™). Cells were maintained in an atmosphere of 5% CO_2_ in a 37 °C humidified incubator.

### Retroviral transduction

HEK 293 T cells were transfected with plasmids encoding VSV-G and Gag-Pol, along with constructs cloned into a retroviral vector (GFP-STRAP, GFP-STRAP ΔC, Myc-TAK1, Myc-p65, shRNA-GFP, or shRNA-STRAP). Media containing viral particles were harvested 48 h post-transfection and filtered through a 0.45-μm membrane. Cells were transduced with the virus by centrifugation at 2,200 rpm for 45 min and incubated for 4 h. Transduced cells were incubated with fresh media for 24 h and then selected with hygromycin or puromycin.

### Reverse transcription-polymerase chain reaction (RT-PCR)

Total RNA from cultured cells was isolated using an RNA prep kit (GeneAll, Seoul, South Korea) and 1 μg of total RNA was reverse transcribed using Moloney Murine Leukemia Virus (MMLV) reverse transcriptase (Enzynomics, Daejeon, South Korea) for 1 h at 42 °C. PCR was performed using the appropriate primers and cDNA, and PCR products were analyzed on an agarose gel containing ethidium bromide. Data were normalized using glyceraldehyde 3-phosphate dehydrogenase (GAPDH) expression.

### Enzyme-linked immunosorbent assay (ELISA)

RAW 264.7 macrophages were seeded in a 24-well plate and treated with LPS (80 ng/ml) or Pam3CSK4 (100 ng/ml). The media were harvested and mouse IL-6, TNF-α, and IL-1β. levels were analyzed using an ELISA kit (BD Biosciences) according to the manufacturer’s recommendations.

### Immunoblot analysis

Cell lysates were prepared in 1% nonyl phenoxypolyethoxylethanol (NP-40) or 1% digitonin with a protease inhibitor cocktail (Roche) and resuspended in denaturing protein loading buffer. Lysates were separated by sodium dodecyl sulfate (SDS)-polyacrylamide gel electrophoresis (PAGE) and transferred to polyvinylidene fluoride (PVDF) membranes (Millipore, Bedford, MA). The membranes were blocked with 5% skim milk in phosphate-buffered saline (PBS) containing 0.1% Tween^®^20 (PBS-T) for 10 min and incubated with the appropriate antibodies at 4 °C overnight. The membranes were washed three times with PBS-T and incubated with horseradish peroxidase-conjugated secondary antibodies for 1 h. Bands were visualized using an enhanced chemiluminescence (ECL) detection reagent (Abclone, Seoul, South Korea).

### Co-immunoprecipitation assay

RAW 264.7 macrophages were stimulated with LPS (80 ng/ml) for the indicated time points and washed twice with PBS. Cells were lysed with 1% digitonin containing a protease inhibitor cocktail (Roche) for 2 h at 4 °C. Cell lysates were incubated with primary antibodies overnight at 4 °C and protein G-Sepharose beads (Sigma-Aldrich) were added to the samples for 1 h at 4 °C. The beads were washed twice with 0.1% digitonin. Proteins were eluted by boiling in denaturing buffer (50 mM Tris-HCl, pH 6.8, 2% SDS, and 5% β-mercaptoethanol) for 5 min.

### Cell fractionation

Cells were lysed in cytosol extraction buffer (10 mM HEPES, pH 7.9, 10 mM KCl, 0.1 mM EDTA, 0.1 mM EGTA, 1 mM DTT, 0.5% NP-40) on ice for 15 min, centrifuged at 4,000 rpm for 10 min, and the supernatant was collected as the cytosolic fraction. The pellet was lysed in hypertonic buffer (20 mM HEPES, pH 7.9, 0.4 M NaCl, 1 mM EDTA, 1 mM EGTA, 1 mM DTT) on ice for 20 min and centrifuged at 13,000 rpm for 20 min and the supernatant was collected as the nuclear fraction.

### Luciferase assay

Cells were seeded in a 12-well plate and transfected with or without STRAP along with the NF-κB (IL-6 promoter) luciferase reporter and Renilla reporter. The cells were then stimulated with LPS for 4 h and lysed with lysis buffer. The luciferase activity was determined using the Dual-Luciferase^®^ Reporter Assay System (Promega, Madison, WI). Firefly luciferase activity was normalized to Renilla luciferase activity.

### Immunofluorescence Assay

Cells were fixed with 3.7% formaldehyde in PBS for 10 min at room temperature and permeabilized with 0.2% Triton™ X-100 in PBS for 10 min. After blocking with 2% bovine serum albumin in PBS (PBA) for 30 min, the samples were incubated with the appropriate primary antibody (1:200) in 2% PBA for 1 h at room temperature. Bound antibody was visualized with Alexa Fluor^®^ 488- or Alexa Fluor^®^ 568-conjugated antibody (1:200, Life Technologies, Carlsbad, CA). DAPI (Sigma-Aldrich) was used as a nuclear counterstain.

### Statistical analysis

All experiments were repeated at least three times, with consistent results. Data are presented as means and standard deviation of the mean, as noted in the figure legends. Statistical differences between two means were evaluated with the two-tailed, unpaired Student’s *t*-test. Differences with *P* values below 0.05 were considered significant. No samples were excluded from the analysis. The data had a normal distribution and the variance was similar between the groups being statistically compared. No statistical method was used to predetermine sample sizes. Sample size was based on previous experience with experimental variability. The experiments were not randomized. The investigators were not blinded to allocation during experiments or outcome assessment.

## Additional Information

**How to cite this article**: Huh, H. D. *et al*. STRAP Acts as a Scaffolding Protein in Controlling the TLR2/4 Signaling Pathway. *Sci. Rep.* **6**, 38849; doi: 10.1038/srep38849 (2016).

**Publisher's note:** Springer Nature remains neutral with regard to jurisdictional claims in published maps and institutional affiliations.
